# Parametrizing the Conditionally Gaussian Prior Model for Source Localization with Reference to the P20/N20 Component of Median Nerve SEP/SEF

**DOI:** 10.3390/brainsci10120934

**Published:** 2020-12-03

**Authors:** Atena Rezaei, Marios Antonakakis, MariaCarla Piastra, Carsten H. Wolters, Sampsa Pursiainen

**Affiliations:** 1 Computing Sciences, Faculty of Information Technology and Communication Sciences, Tampere University, Hervanta Campus, P.O. Box 1001, 33014 Tampere, Finland; sampsa.pursiainen@tuni.fi; 2 Institute of Biomagnetism and Biosignalanalysis, University of Münster, Malmedyweg 15, D-48149 Münster, Germany; marios.antonakakis@uni-muenster.de (M.A.); mariacarla.piastra@donders.ru.nl (M.P.); carsten.wolters@uni-muenster.de (C.H.W.); 3 Cognitive Neuroscience, Donders Institute for Brain, Cognition and Behaviour, Radboud University Nijmegen Medical Centre, Kapittelweg 29, 6525 EN Nijmegen, The Netherlands; 4 Otto Creutzfeldt Center for Cognitive and Behavioral Neuroscience, University of Münster, 48149 Münster, Germany

**Keywords:** electroencephalography (EEG), magnetoencephalography (MEG), somatosensory evoked potentials, somatosensory evoked fields, P20/N20 component, hierarchical bayesian model, parametrization, deep activity

## Abstract

In this article, we focused on developing the conditionally Gaussian hierarchical Bayesian model (CG-HBM), which forms a superclass of several inversion methods for source localization of brain activity using somatosensory evoked potential (SEP) and field (SEF) measurements. The goal of this proof-of-concept study was to improve the applicability of the CG-HBM as a superclass by proposing a robust approach for the parametrization of focal source scenarios. We aimed at a parametrization that is invariant with respect to altering the noise level and the source space size. The posterior difference between the gamma and inverse gamma hyperprior was minimized by optimizing the shape parameter, while a suitable range for the scale parameter can be obtained via the prior-over-measurement signal-to-noise ratio, which we introduce as a new concept in this study. In the source localization experiments, the primary generator of the P20/N20 component was detected in the Brodmann area 3b using the CG-HBM approach and a parameter range derived from the existing knowledge of the Tikhonov-regularized minimum norm estimate, i.e., the classical Gaussian prior model. Moreover, it seems that the detection of deep thalamic activity simultaneously with the P20/N20 component with the gamma hyperprior can be enhanced while using a close-to-optimal shape parameter value.

## 1. Introduction

This article concerns computational source localization methods for the activity of the brain in electro- and magnetoencephalography (EEG and MEG) [1,2,3]. Reconstructing the primary current density of the neurons as a three-dimensional (3D) distribution restricted to the grey matter is an ill-posed inverse problem, in which the prior model and reconstruction technique applied have a major effect on the final result [3]. Consequently, *a priori* information, such as an anatomically and physiologically accurate head model, is needed. When the inverse problem is formulated via Bayesian statistics, the *a priori* knowledge can be rigorously modelled as a statistical prior distribution [4,5,6,7,8].

EEG and, particularly, MEG, are famously known to be more sensitive to the superficial parts than to the deeper lying areas, e.g., the thalamus [9,10]. In this study, we focused on developing the conditionally Gaussian hierarchical Bayesian model (CG-HBM) [11], which, based on numerical simulations [4,5,6,7,8], has been suggested as a potential approach to reconstructing networks of focal sources with variable depth. In CG-HBM, the prior has a hierarchical structure; the variance of a Gaussian conditional prior is steered by a heavy-tailed hyperprior. This allows the primary current density to have a considerably greater focal amplitude when compared to the background fluctuations than what is otherwise possible with a Gaussian prior. CG-HBM forms a superclass for different inversion methods as well as a potential platform for the development of new source localization methods [12]. In clinical applications, focal reconstructions are needed, e.g., in epileptic focus localization and in the analysis of epileptic networks during seizures for adults and paediatrics [13,14,15].

The aim and novelty of this proof-of-concept study is to improve the applicability of the CG-HBM for localizing sources with variable depth by proposing a simple and robust parametrization approach that has been designed to remain invariant with respect to alterations in the noise level [10,16,17,18] and the size of the source space [5,19,20], with both these factors being essential with respect to the localization outcome. An important goal is to obtain an appropriate localization performance with different hypermodels and reconstruction techniques. Namely, one of the major challenges in using CG-HBM is that the mutual differences between these models and techniques can be significant, if the model parameters are not optimally set [6,7]. We referred to both simulated and experimental somatosensory evoked potential (SEP) and field (SEF) datasets, and then selected the parameter values with the aim of detecting the activity corresponding to the P20/N20 component, i.e., the 20 ms post-stimulus response, occurring in the median nerve (wrist) stimulation [21,22,23,24]. The Brodmann area 3b activity in the hand-knob of the primary somatosensory cortex was reconstructed with a high-density forward model. This stable and transient activity generally has an excellent signal-to-noise ratio (SNR), especially in MEG, but also in EEG [22,25] and it is, therefore, well-suited for finding a high-density reconstruction. Additionally, a sparse model [19] was applied in order to detect the deep thalamic activity that is associated with the P20/N20 response.

We compared the gamma (G) and inverse gamma (IG) hyperprior in detecting the activity in these regions. The previous numerical simulation studies [7,12] suggest that G and IG can lead to two characteristically different reconstructions, if the shape and scale parameter, i.e., β and the $\theta_{0}$ determining the hyperprior are not ideally set. In particular, the shape parameter value β=1.5 results in a suppressed deep activity with G hyperprior, as shown in [12]. We minimized the posterior difference between G and IG by optimizing β, while an initial range for $\theta_{0}$ was obtained based on the prior-over-measurement signal-to-noise ratio (PM-SNR), i.e., the relative weight of the prior compared to the measurement noise. Here, we introduce PM-SNR as a new concept to allow for balancing the $\theta_{0}$-value with respect to the source space size and the estimated level of the measurement and modeling errors. The initial value for PM-SNR can be related to the existing knowledge of the Tikhonov-regularized minimum norm estimate (MNE) [26,27] following from the classical Gaussian prior model [28].

In our experiments, the iterative alternating sequential (IAS) and Markov chain Monte Carlo (MCMC) sampling techniques presented in [7,29,30] were applied in order to reconstruct the activity. We used a finite element method (FEM) that is based on the forward modeling approach [31,32], which allows for creating an accurate volumetric discretization of a multi-compartment head segmentation regarding its conductivity distribution and strongly folded tissue structures [33,34,35,36,37]. The source localization experiments were performed while using the *Zeffiro* interface (the source code is available online at https://github.com/sampsapursiainen/zeffiro_interface) (ZI) software pipeline [38], which couples the FEM forward model with CG-HBM.

The results that were obtained for the known synthetic source suggest that, through *maximum a posteriori* (MAP) estimation, one can reconstruct a simulated P20/N20 component without *a priori* limiting the region of the activity. It was observed that MCMC sampling allowed for the posterior to be adopted to the structure and resolution of the underlying numerical model and geometry, thus avoiding numerical bias, e.g., overly focal results. We compared our CG-HBM reconstructions to Tikhonov-regularized MNE [1] and the minimum current estimate (MCE) [39], which can be interpreted as special cases of the IAS method in combination with the G hyperprior and with β=1.5. Based on our findings for three subjects, we suggest that, by choosing an optimization-based shape parameter value β=3 and a PM-SNR of 0–30 dB, with the exact value being determined by the modeling accuracy assumed, the cortical generator of the P20/N20 component can be localized in the Brodmann area 3b with both simulated and measured data. Moreover, it seems that the detection of the correlated sub-cortical thalamic activity, simultaneously with the cortical one, could be enhanced using the close-to-optimal shape parameter value, when the G hyperprior was used.

## 2. Methods

This section briefly reviews the mathematical CG-HBM approach and its implementation in this study. The primary current density is denoted by x, which is the discretized approximation of the primary current density $|{\vec{J}}^{p}|$ in the brain, i.e., the unknown of the inverse problem, and the measurement data vector is represented by y. In both EEG and MEG, the dependence of y on x, i.e., the forward model can be formulated via the lead field matrix equation of the form y=Lx+n, where n is a noise vector and L is the so-called lead field matrix [1]. Here, L is obtained via the FEM discretization of the classical field equations following from the quasi-static approximation of the Maxwell equations, as described in [31,40,41]. For the generality of the presentation, we assume that L is obtained using SI-units, while y and **n** are normalized with respect to the amplitude *A* (here, the $\ell^{2}$-norm) of the measured or simulated signal.

### 2.1. Conditionally Gaussian Hierarchical Bayesian Model

For a single given dataset y, the classical Bayes formula for subjective conditional probabilities can be written as

(1) $$p\left(x|y\right)=\frac{p(x)\;p(y|x)}{p(y)}\propto p(x)\;p(y|x).$$

That is, the *posterior* probability density p(x∣y) of the unknown discretized primary current density x in the brain is proportional to the product between the prior density p(x), i.e., the *a priori* knowledge of x, and the likelihood function p(y∣x) that follows from the measurement noise model [42].

The measurement error is assumed here to be a Gaussian zero mean random vector n=y−Lx with independent entries. Consequently, the likelihood is of the form $p\left(y|x\right)\propto \exp(-{\left(2\sigma^{2}\right)}^{-1}∥Lx-y{∥}^{2})$, where σ is the standard deviation of the noise. In the hierarchical Bayesian approach, one assumes the prior to be a joint density p(x,θ)∝p(θ)p(x∣θ) of x and a hyperparameter θ. That is, the posterior is of the form

(2) p(x,θ∣y)∝p(θ)p(x∣θ)p(y∣x).

In CG-HBM [7,11,43], the conditional part p(x∣θ) is also a zero mean Gaussian density. Its diagonal covariance matrix is predicted by a heavy-tailed hyperprior p(θ), which means that the variance vector, i.e., the set of diagonal entries, is likely to contain outliers. Thus, it is implicitly assumed that x is a sparse vector with a small subset of entries, which are noticeably large in absolute value when compared to the other entries [6]. The number and intensity of these outliers are controlled by the hyperprior [43]. The resulting impulse-like prior model for the unknown is particularly useful in obtaining a focal reconstruction for the brain activity. The parameters determining the hyperprior allow for the level of the focality to be tuned, i.e., the rough relative portion of the *x*-entries that are likely to *a priori* differ from zero.

#### 2.1.1. Posterior Exploration

Given the posterior, the actual reconstruction can be found via several different approaches. The most common ones can be divided into optimization and sampling techniques. The former include the MAP algorithms, which are aimed at finding the maximizer of the posterior density, i.e., $x_{MAP}=\mathrm{argmax}\;p_{\mathrm{post}}\left(x,\theta |y\right)$. MAP estimation usually provides a faster, but less robust, way to obtain a reconstruction than the sampling techniques, e.g., MCMC methods, which approximate the conditional mean $x_{CM}=E\left(x,\theta |y\right)=\int \left(x,\theta \right)p_{\mathrm{post}}\left(x,\theta |y\right)\;dx\;d\theta$ [28]. MCMC methods generate samples from the posterior distribution by constructing a Markov chain that has the target posterior distribution as its equilibrium distribution. A more detailed descriptions for the IAS MAP estimation method and the MCMC sampler that was employed for conditional mean (CM) estimation can be found in [7].

#### 2.1.2. Gamma and Inverse Gamma Hyperprior

As the hyperprior, we use both the gamma $G(\theta |\beta ,\theta_{0})$ and inverse gamma $\mathrm{IG}(\theta |\beta ,\theta_{0})$ distributions, whose densities are supported on the set of non-negative real numbers with a structure that is determined by the scale and shape parameter $\theta_{0}$ and β, respectively. In the present approach, the scale parameter $\theta_{0}>0$ essentially sets the expected variance of the conditionally Gaussian prior. It can be interpreted as the capability of the prior to detect brain activity growing along with the value of $\theta_{0}$. The shape parameter steers the rate of the decay for the tail part. Finding a suitable value for $\theta_{0}$ and β is essential in avoiding an over- or under- sensitive prior, which might involve depth-bias or emphasized noise-effects. Based on our earlier experience, this is especially important with regard to G hyperprior, which might suppress deep activity, when β=1.5 [12]. In order to make both the G and IG hyperpriors perform similarly given $\theta_{0}$, we select β to be uniformly β=3, which can be interpreted as a close-to-optimal choice for minimizing the differences between the outcomes of G and IG (Appendix A).

#### 2.1.3. Total Scale

The source-wise scale parameter $\theta_{0}$ can be adapted to a given forward model by introducing a total scale $\theta_{0}^{\left(\mathrm{tot}\right)}$, which gives the scale per distribution, while $\theta_{0}$ represents the scale per source. This follows from the additivity of the Gaussian prior variance, i.e., that the nearby sources $s_{1}$ and $s_{2}$ with variances $\theta_{1}$ and $\theta_{2}$ have the total variance $\theta_{1}+\theta_{2}$, if interpreted as a single source $s=s_{1}+s_{2}$. Thus, the relationship between the total and source-wise scale parameter can be written as

(3) $$\theta_{0}=\frac{\theta_{0}^{\left(\mathrm{tot}\right)}\sigma^{2}A^{2}}{N}\;\mathrm{or}\;\theta_{0}^{\left(\mathrm{tot}\right)}=\frac{\theta_{0}N}{\sigma^{2}A^{2}}$$

Namely, to have an invariant weight with respect to the likelihood, the scale $\theta_{0}^{\left(\mathrm{tot}\right)}$ of the conditionally Gaussian prior must be directly proportional to the source position count *N* of the forward model and inversely proportional to the relative noise variance $\sigma^{2}$ and the squared amplitude $A^{2}$. Because we assume $\theta_{0}^{\left(\mathrm{tot}\right)}$ to be an application-specific constant, we introduce here, as a new concept, the following prior-over-measurement signal-to-noise ratio

(4) $$\mathrm{PM}-\mathrm{SNR}\;=\;\mathrm{dB}\left(\sqrt{\theta_{0}^{\left(\mathrm{tot}\right)}}\right)\;=\;\mathrm{dB}\left(\sqrt{N}\right)\;+\;\mathrm{dB}\left(\delta \right)\;+\;\mathrm{dB}\left(A\right)\;-\;\mathrm{dB}\left(\delta^{\left(\mathrm{ref}\right)}\right)\;\;\;\mathrm{with}\;\delta \;=\;\frac{\sqrt{\theta_{0}}}{\sigma A}\;,\;\;\;\delta^{\left(\mathrm{ref}\right)}\;=\;\frac{|{\vec{J}}^{p}|}{A}\;,$$

and $\mathrm{dB}\left(x\right)=20\log_{10}x$. Here, $\delta^{\left(\mathrm{ref}\right)}$ is a reference level which is obtained as the ratio between the *a priori* estimated norm of the primary current density $|{\vec{J}}^{p}|$ and the signal amplitude *A*. The term dB(A) is included in (4), as we present $\theta_{0}$ and $\theta_{0}^{\left(\mathrm{tot}\right)}$ with respect to normalized amplitude A=1. PM-SNR measures the relative weight of the prior as compared to the noise level and it is balanced by the system size. At the reference level, when PM-SNR is 0 dB or $\delta \sqrt{N}=\delta^{\left(\mathrm{ref}\right)}$, $\sqrt{\theta_{0}}$ matches the *a priori* noise-induced fluctuation of the candidate solution normalized by $A\sqrt{N}$, which is, $\sqrt{\theta_{0}}=\sigma |{\vec{J}}^{p}|/\left(A\sqrt{N}\right)$. In order to generalize these expressions for the case of a non-diagonal noise covariance, one can interpret the relative noise variance $\sigma^{2}$ as the largest eigenvalue, i.e., the $\ell^{2}$-norm, of the noise covariance matrix. The PM-SNR of the scale parameter values (Table 1) that were applied in this study refer to the present EEG amplitude A= 10^−6^ V that was obtained as the $\ell^{2}$-norm of the measured P20/N20 component, relative noise standard deviation of σ=0.03, and the typical 10 nAm dipolar source strength in the brain [1,44,45], i.e., $|{\vec{J}}^{p}|=$$10^{-8}$ Am.

Because δ also represents the relative weight of a non-conditional Gaussian prior, it can be associated with the Tikhonov regularization parameter of MNE [7,26,28]. In the Brainstorm software, the default level of the relative prior weight is set to a constant value dB(δ)= 9 dB, as shown in the tutorial [27]. Taking into account the source count of this tutorial, which is 15,000, this would result in a PM-SNR of 10 dB. However, notice that a constant default level is not invariant under changing the dimension of the source space.

Note that the current definition (4) of PM-SNR has been selected in order to obtain a uniform representation with respect to G and IG hyperprior. Alternatively, PM-SNR can be defined with respect to the mean of the hyperprior that is given by the total scale, i.e., $\theta_{0}^{\left(\mathrm{tot}\right)}\beta$ and $\theta_{0}^{\left(\mathrm{tot}\right)}/\left(\beta -1\right)$ for G and IG, resulting in a correction of $+\mathrm{dB}(\sqrt{\beta })=+5$ dB and $-\mathrm{dB}(\sqrt{\beta -1})=-3$ dB, when β=3, respectively.

#### 2.1.4. Latent Noise Effects

We assume that the relative (total) noise standard deviation is of the form $\sigma =s\bar{\sigma }$, where $\bar{\sigma }$ is the standard deviation of the *a priori* known noise and s≥1 is a correcting term due to latent noise effects, such as forward modeling inaccuracies due to the quasi-static approximation, inter-individual head tissue conductivity differences, and/or segmentation errors, see e.g., [46]. Denoting $\bar{\delta }=\sqrt{\theta_{0}}/\left(\bar{\sigma }A\right)$, it follows that the PM-SNR is of the form

(5) $$\mathrm{PM}-\mathrm{SNR}=\bar{\mathrm{PM}-\mathrm{SNR}}+\mathrm{dB}\left(s\right),$$

where dB(s) is the total contribution of the latent noise effects. Thus, PM-SNR will be greater than $\bar{\mathrm{PM}-\mathrm{SNR}}$ for models including latent noise, i.e., when s>1: the greater the latent noise, the greater weight of the prior. When $\bar{\mathrm{PM}-\mathrm{SNR}}$ is 0 dB, PM-SNR is given by dB(s) and the explicit formula for the scaling parameter is $\sqrt{\theta_{0}}=s\sigma \left|{\vec{J}}^{p}\right|/\left(A\sqrt{N}\right)$, where σ corresponds to the known noise level and *s* to the *a priori* assumption of the latent noise strength.

The shape parameter choice defines the spread of the hyperprior that can be related to the uncertainty of $\delta^{\left(\mathrm{ref}\right)}$ following from, e.g., the potentially varying source depth that affects the amplitude of the measurement: the smaller the shape parameter value, the greater the spread. With the current value β=3, the interdecile range (Appendix B) of the hyperprior p(θ) obtains values from 0.4E(θ) to 1.8E(θ) with E(θ) denoting the hyperprior mean. Thus, $\sqrt{\theta }$, i.e., the expected source strength given θ, varies $\mathrm{dB}(\sqrt{1.8/0.4})=7$ dB over the interdecile range. This matches our approximation for the variation of $\delta^{\left(\mathrm{ref}\right)}$ that was obtained as the interquartile range of the lead field based simulated $\ell^{2}$-norm signal amplitude over the source space.

### 2.2. Numerical Model

#### 2.2.1. Head Segmentation

The FE implementation approach that was presented in [47] was applied, including the formula for the EEG and MEG lead field matrix. A tetrahedral finite element mesh was generated by subdividing each voxel in a surface-based regular hexahedral segmentation into six tetrahedra. The FE meshes were generated while using a six-layer surface segmentation based on T1-weighted and T2-weighted MRI sequences recorded with a 3T MRI scanner. The surfaces (level-sets) of skin, compact bone (skull), spongious bone (skull), cerebrospinal fluid (CSF), grey matter, and white matter were included in the model. An FE mesh was generated for both 1 and 2 mm resolution (voxel size). The first of these included 3.8 M nodes and 22 M tetrahedra and the second one 0.47 M nodes and 2.7 M tetrahedra.

#### 2.2.2. Source Space

In order to generate the source space of x, we used the FEM-based quadratic H(div) approach presented in [31], employing the Position Based Optimization (PBO) interpolation with the 10-source (eight-point) stencil. That is, a given dipolar current source was estimated via the four linear face and six quadratic edge-based vector basis functions that are associated with the barycenter of the tetrahedron containing the source position. The sources were placed in the interior part of the grey matter compartment in the elements with a full set (four) of neighbors belonging to the same compartment. The rest of the compartment forming the boundary layer of the grey matter contained no sources, since the modeling accuracy is known to be reduced for the boundary layer [40].

A total of $10^{5}$ points were distributed randomly in the grey matter for each FE mesh in order to obtain a uniform (mesh-independent) source density for reconstructing the somatosensory 3b activity. This initial source count was selected to allow the present source localization accuracy, in principle, to surpass the *a priori* known maximum limit of EEG and MEG, which is about 9 mm and 2–4 mm, respectively [22,48,49,50,51]. A uniform point spread was obtained through a straightforward random permutation due to the uniform mesh structure.

**Figure 1:**
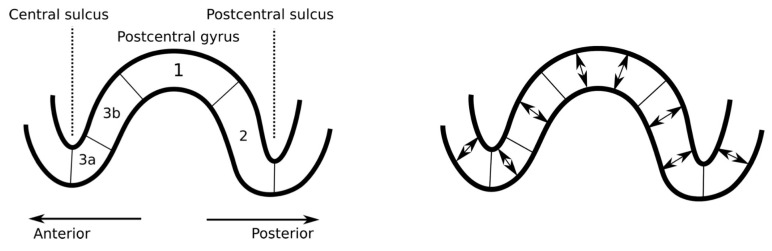
**Left:** a schematic illustration depicting the sagittal cut of the primary somatosensory cortex. The P20/N20 component of the somatosensory activity occurs in the Brodmann area 3b, which is located in the posterior wall of the central sulcus [24]. **Right:** the orientation of the primary currents (somatosensory evoked potential (SEP)/somatosensory evoked field (SEF) components) in the cerebral cortex is normal with respect to the surface due to the normal alignment of the pyramidal cells [10].

The points that were placed on the boundary layer in the initial stage were filtered out of the eventual distribution, which consisted of $7.6\times 10^{4}$ and $6.1\times 10^{4}$ positions for the 1 and 2 mm FE meshes, respectively. The slightly lower source count for the 2 mm case was caused by the thicker boundary layer that arose from the larger element size. Each position comprised three sources that were oriented along the three Cartesian coordinate axes. Hence, the total number of sources was $2.28\times 10^{5}$ and $1.83\times 10^{5}$ for the 1 and 2 mm FE meshes, respectively. The source localization experiments were primarily conducted with the 1 mm resolution, while the 2 mm accuracy was utilized in order to examine the forward modeling effects on the source localization. In addition to the dense source space, a sparse one was created to enhance the detectability [19] of the thalamic component simultaneously with the Brodmann area 3b activity [21,52,53]. In the sparse distribution, the number of source positions was 1/100 as compared to that of the dense one and so the 1 mm mesh was used in the forward simulation. The Cartesian set of sources was used in inverting the data. After the inversion process, the distribution obtained was projected in the cortical areas while using the normal constraint (Figure 1) of the cortex. In other words, the vector field component parallel to the surface normal constituted the final reconstruction. In the sub-cortical areas, the normal projection was not applied, as the sub-cortical neurons are not generally oriented along the surface normal of the neuronal tissue.

### 2.3. Measured Data

**Figure 2:**
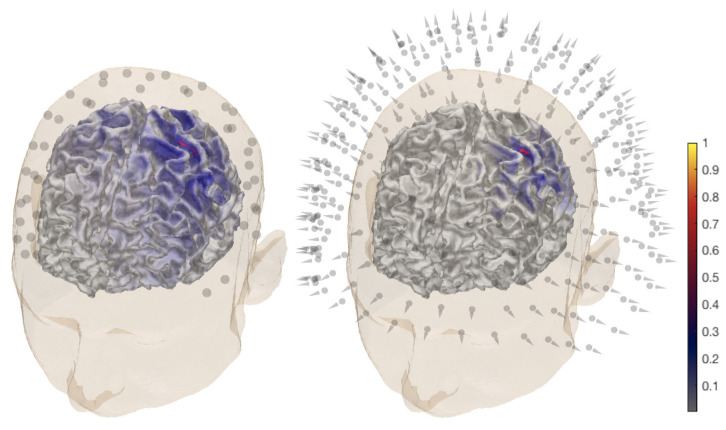
General overview of the P20/N20 component reconstruction for subject (I). The activity is found in the posterior bank of the central sulcus, the Brodmann 3b area for electroencephalography (EEG) (**left**) and magnetoencephalography (MEG) dataset (**right**). A MAP estimate of the global source distribution is visualized on the surface of the white matter. The locations of 72 EEG electrodes and 271 magnetometers are shown in the left and right images, respectively.

**Figure 3:**
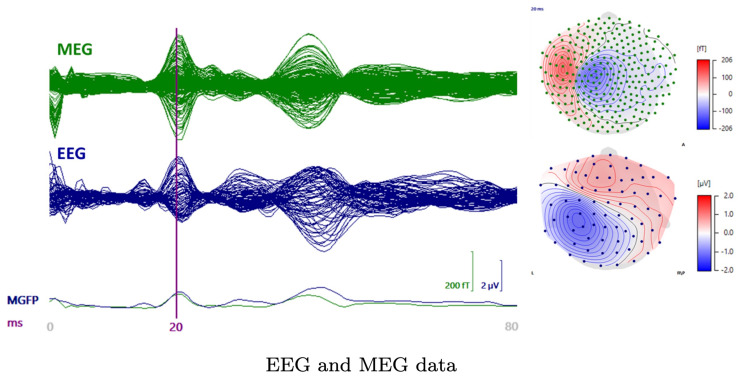
A butterfly plot of the somatosensory evoked field (SEF) (upper left) and potential (SEP) (lower left) from 0 to 80 ms post-stimulus with the 20 ms time point being indicated by the vertical line. The P20/N20 peak topographies for SEF (upper, right) and SEP (lower right) are also visualized.

The source localization experiments were conducted while using a dataset that was obtained for three healthy and right-handed adult male subjects (I), (II), and (III), who were 49, 32, and 27 years old, respectively. The right median nerve was stimulated with the subject lying in a supine position in a magnetically shielded room. Simultaneous SEP/SEF measurements were performed while using 80 AgCl sintered ring electrodes (EASYCAP GmbH, Herrsching, Germany), including 74 EEG channels with an additional six channels for detecting eye movements together with an MEG setup (OMEGA2005, VSM MedTech Ltd). Four out of a total of 275 magnetometers and two out of 74 EEG sensors were reported as defective channels. Therefore, the measurements from 72 electrodes and 271 magnetometers (Figure 2) were used in the eventual dataset. A total of 1200 stimuli were obtained during a 10 min. measurement session. The electric pulse duration was 0.5 ms. In order to determine the magnitude, the stimulus strength was increased until a clear movement of the thumb was visible. Each measurement had a 300 ms total duration, which was subdivided into a 100 ms pre-stimulus and 200 ms post-stimulus sub-interval. The inter-stimulus interval varied between 350 and 450 ms to avoid habituation. The measurements were averaged and pre-processed while using a notch filter for the 50 Hz frequency and its harmonics to remove the power-line noise. The responses measured for the different stimuli were averaged to produce the SEP/SEF dataset (Figure 3) the amplitude of which was normalized to one.

The data were filtered while using a bandpass of 20–250 Hz [52]. The data vector y for the inversion computation corresponded to the P20/N20 activity peak occurring at the 20 ms post-stimulus time point (Figure 3).

### 2.4. Synthetic Data

**Figure 4:**
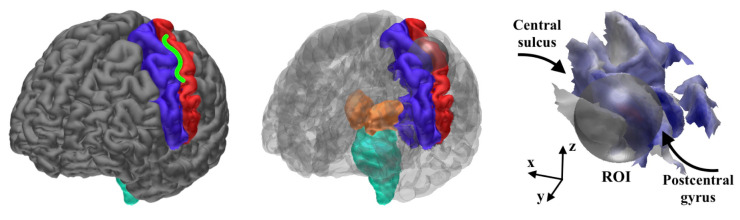
(**Left**) the hand knob (green Ω shape) is a part of the Brodmann area 3b in the central sulcus (between the pre-central (blue) and post-central (red) areas) [10]. (**Center**) regions of interest (ROIs) for the CM computations; the spherical region of interest (ROI) for detecting the 3b activity in the hand-knob together with the ROI for the thalamic activity detection, including the post-central area (red), thalamus (orange), and brainstem (cyan). (**Right**) the 24 mm diameter spherical ROI (grey) approximately covers the hand-knob of the left hemisphere. A global MAP estimate for the synthetic EEG data is visualized on the cortex.

In order to enable a comparison between the measured and synthetic data, a normally-oriented synthetic dipolar source was placed in the hand-knob of the 3b area in the posterior wall of the central sulcus (Figure 4). Its position in the MNI (Montreal Neurological Institute) coordinate system was x=−38mm, y=−28mm, z=56mm with ±2 mm accuracy. The data for the source were simulated while using the present FEM-based forward model and additive zero mean Gaussian noise with 3% standard deviation with respect to the maximal signal amplitude. The same noise model was also applied in order to obtain the likelihood function for the measured data.

### 2.5. Source Localization Estimates

The source localization tests were performed using the dense and sparse source distributions (Table 1) that are described in Section 2.2.2. The first of these was applied to be as accurate as possible in detecting the cortical source of the P20/N20 component in the 3b area using both measured and synthethic data. The reason for using the sparse distribution was to enhance the detectability of the thalamic activity [19], which occurs simultaneously with the cortical peak [52,53].

The MAP estimate was found via three IAS iteration steps. For CM, a sample of 10,000 points was created with the MCMC sampler. Of these, 1000 points in the beginning of the sequence were neglected as a burn-in phase. When examining the 3b area, the MCMC-based CM evaluation was due to the high dimensionality of the source space, performed by limiting the activity within a relatively small spherical ROI that was defined based on the MAP estimates (Figure 2). The placement of the ROI (Figure 4) was selected according to the literature on the hand-knob within the Brodmann area 3b [10]. The difference vector pointing from the synthetic source position to the center point of the ROI was Δx=4mm, Δy=−1mm, Δz=2mm. Rather than being regarded as a complete reconstruction approach, the spherical ROI is primarily regarded here as a tool for analyzing the peak of the posterior in the vicinity of the area where the activity is maximized. When reconstructing the thalamic source via MCMC sampling, the ROI covered the somatosensory area together with the sub-cortical thalamus and brainstem structures (Figure 4). In order to investigate the effects of the measurement noise, the P20/N20 response was reconstructed with G hyperprior by averaging the EEG data for 300 and 100 epochs. When compared to the principal case of 1200 epochs, this can be estimated to lead to +6 dB and +10 dB increments of the relative measurement noise level σ, based on the central limit theorem, i.e., $\mathrm{dB}(\sqrt{1200/300})\approx +6\mathrm{dB}$ and $\mathrm{dB}(\sqrt{1200/100})\approx +10\mathrm{dB}$. In the case of elevated noise, the scale parameter was adjusted according to (4), while assuming that PM-SNR is unaffected by any change in σ. Moreover, MNE and MCE were investigated as alternative reconstruction approaches for EEG. These were obtained as the first and third step iterates of the IAS algorithm while using the G hyperprior with β=1.5, i.e., the value that yields the match between IAS, MNE, and MCE [7].

#### Implementation in Zeffiro Interface

The present forward and inverse methods have been implemented in the *Zeffiro* interface (ZI) [38] toolbox, which uses the Matlab (The MathWorks Inc., Natrick, MA 01760, USA) platform. ZI aims to provide a user-friendly tool for advanced forward and inverse computations, e.g., accurate lead field matrix construction, source localization, and time-lapse data analysis. ZI’s on-line code repository (see Introduction) includes the methods that were used in the present study. To speed up the processing, ZI utilizes a Graphics Processing Unit (GPU) in the following processes: (1) segmenting the FE grid, (2) creating the lead field, (3) source space interpolation for visualizing the reconstructions, and (4) inverting the data. The following computation times were obtained for a 1 mm resolution six-compartment test mesh with 36 M elements, 6 M nodes, and 0.5 M sources while using a Lenovo P910 ThinkStation (Lenovo, Hong Kong, China) that was equipped with 2 × Intel Xeon E5-2697A v4 CPUs (Intel, Santa Clara, CA 95054, USA)(RAM 256 GB) and 2 ×NVIDIA Quadro P6000 GPUs (NVIDIA, Santa Clara, CA 95051, USA)(RAM 24 GB): (1) FE mesh generation = 1329 s, (2) EEG lead field for 128 electrodes = 2362 s, (3) MEG lead field for 154 magnetometers = 4826 s, and (4) Source space interpolation = 212 s.

## 3. Results

**Figure 5:**
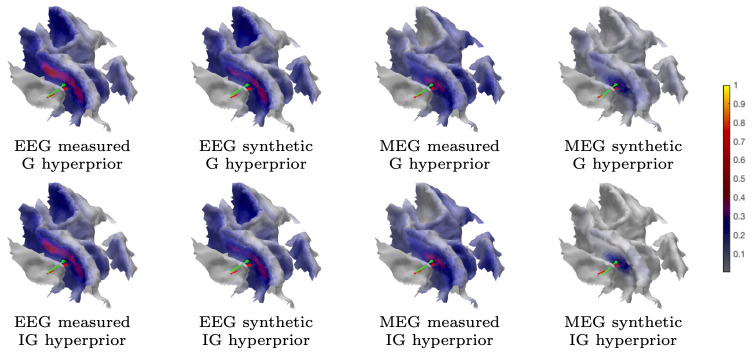
Subject (**I**). The MAP estimation results obtained with the iterative alternating sequential (IAS) iteration corresponding to the measured and synthetic data and the 1 mm FE mesh. The placement and orientation of the synthetic source is shown by the red pin and the mass center of the reconstruction by the green one. The MAP estimates shown have been obtained using a global source space.

**Figure 6:**
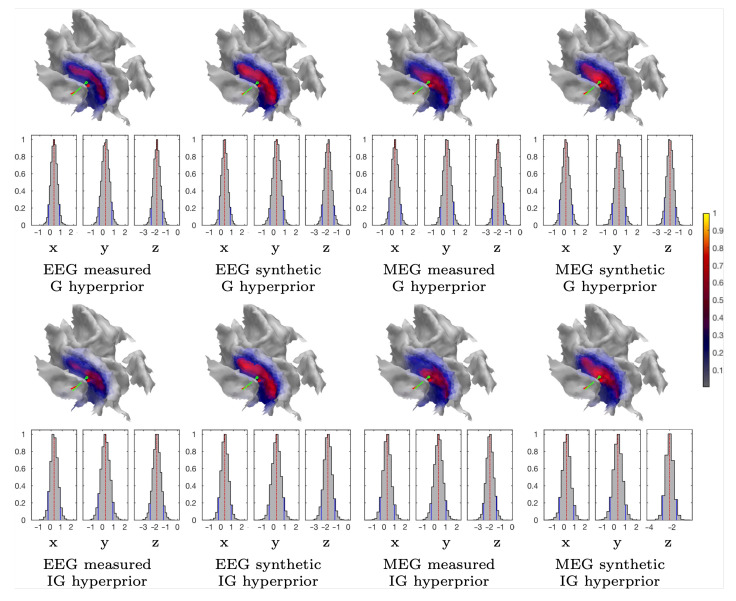
Subject (I). The CM estimation results that were obtained with the sampler corresponding to the measured and synthetic data and the 1 mm FE mesh. The marginal density (histograms) of the (volumetric) posterior mass centre is illustrated for each case and the Cartesian coordinate component, including the median (red dashed line) and the 90% credibility interval, is conditional on the subjectively selected parameters (solid blue line). The placement and orientation of the synthetic source is shown by the red pin and mass center of the reconstruction by the green one. The CM estimates shown have been obtained by limiting the source space within ROI.

**Figure 7:**
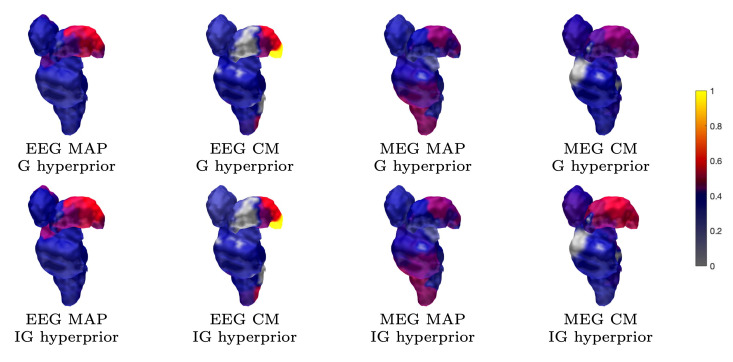
Subject (I). The thalamic activity found with 1200 epochs and 1 mm FE mesh resolution. The ventral posterolateral part of the left thalamus (contralateral to the stimulation side) can be observed as having been activated in most reconstructions. The source space is global for the MAP estimates and limited to ROI in the case of CM.

**Figure 8:**
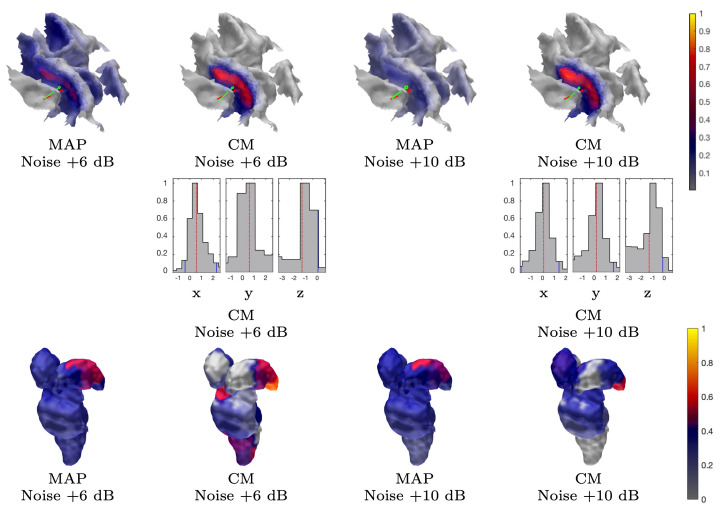
Subject (I). The results obtained with an EEG and G hypermodel while using 300 and 100 epochs, i.e., approximately +6 dB and +10 dB noise level, respectively. The source space is global for the MAP estimates and limited to ROI in the case of CM.

**Figure 9:**
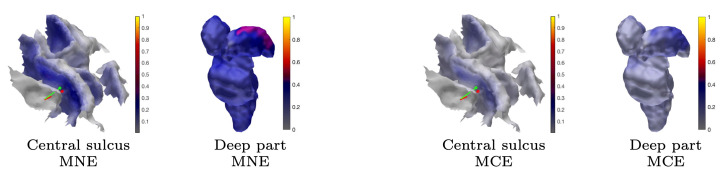
Subject (I). Minimum norm estimation (MNE) and minimum current estimation (MCE) results for the P20/N20 component network for surface activity (central sulcus) and deep activity (thalamus). The source space is global for both MNE and MCE.

**Figure 10:**
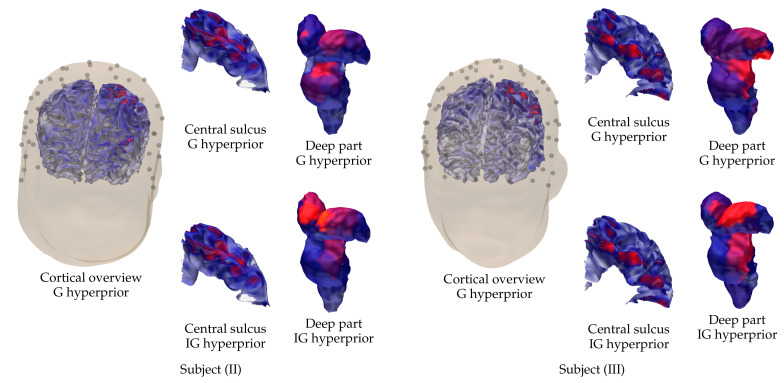
Visualization of the cortical and sub-cortical activity reconstructed for subjects (II) on the left and (III) on the right using the EEG data corresponding to the P20/N20 component. In addition to the cortical overview, showing the reconstruction that was obtained with G hyperprior, excerpts of the cortical activity around the central sulcus and the sub-cortical (deep) activity are shown for G and IG hyperprior.

This section describes the results that were obtained with the data of subjects (I)–(III). An in-depth source localization analysis was conducted in the case of (I). Additionally, the parameters that were suggested by this analysis were tested with (II) and (III) in order to learn about the inter-subject variability of the results. The results of the source localization analysis have been included in Figure 5, Figure 6, Figure 7, Figure 8, Figure 9 and Figure 10 and Table 2 shows the results of the additional tests.

### 3.1. Subject (I)

The proposed parametrization approach was found to perform appropriately in detecting the peak of the P20/N20 component in the area 3b of the left hemisphere. In addition to the activity of the 3b area, the overlaid thalamic component was found to be detectable in the experiments that were performed with the sparse source density. In each source localization test, the scale parameter value was selected based on PM-SNR, whose case-specific value was within the range 0–30 dB depending on the assumption of the latent noise level (Section 2.1.4): the greater the latent noise, the larger the PM-SNR, i.e., the stronger the prior. PM-SNR was chosen to be 20 dB and 0 dB for the measured and synthetic data, respectively, when assuming that the latent modeling errors that are related to the measured data are emphasized by a +20 dB difference in PM-SNR as compared to the simulated case. The PM-SNR value for the MAP estimation process was set to be larger than for the CM evaluation, allowing +10 dB for the latent errors due to the *a priori* lower accuracy of the MAP as compared to CM [28], if the gradient-based IAS algorithm did not otherwise find the peak of the posterior. An important criterion in selecting PM-SNR was the shape of the posterior density, whose peak in the 3b area was assumed to be a few millimeters diameter w.r.t. the mass center of x, i.e., comparable to the mutual distance of the lead field source space density. This was done in order to avoid the failure of the sampling process due to an excessively peaked posterior structure.

#### 3.1.1. Brodmann Area 3b

Table 2 includes a numeric measure for the spread (focality) of the reconstructed and normally restricted activity (in the 3b area), defined as the area of the set, in which its intensity exceeds 80% of the maximum value. The position and orientation differences between the synthetic source and mass center of the reconstructed activity are also given. In the case of the measured data, the synthetic source is regarded as a numerical reference point and it should not be confused with the actual activity (ground truth). Figure 5, Figure 6, Figure 8, and Figure 9 visualize the reconstructed activity on the white matter surface (i.e., on the inner surface of the grey matter) in the vicinity of the ROI. Each distribution shows the activity in the direction of the outward-pointing surface normal.

#### 3.1.2. MAP Estimation

The MAP estimates obtained for the 3b area are shown in Figure 5, Figure 8, and Figure 9. The CG-HBM estimates obtained using 1200 epochs generally localize the activity in the sulcal wall with the difference in position and orientation being less than 0.4 mm and 10.4 degrees. In the 1 mm case, they were a maximum 3.8 mm and 14.4 degrees, respectively. MEG provided slightly more focal reconstructions than EEG, and the IG hyperprior led to more focal outcomes than G for three out of the four reconstructions. The 1 mm FE mesh resolution yielded a greater similarity between the MAP estimates obtained with both the measured and synthetic data than the 2 mm mesh did, as shown by Table 2. In the 2 mm case, the reconstructions were less intense and more spread out than those that were obtained with the 1 mm mesh. The estimates obtained with 300 and 100 epochs (Figure 8), i.e., with +6 dB and +10 dB noise levels, show that the distinguishability of the 3b activity decreases as the noise increases as; the activity is clearly visible in the +6 dB case, but barely detectable with +10 dB noise. The estimates that were obtained with MNE and MCE (Figure 9) show a clear difference in the 3b area, suggesting that the stronger tendency of the MCE to find a focal estimate results in a clearer source distinction than with MNE, while the peak of both estimates is less intense than with the CG-HBM estimates.

#### 3.1.3. CM Estimation

Figure 6 and Figure 8 illustrate the CM estimates that were obtained for the spherical ROI together with the corresponding marginal densities (histograms) for the volumetric mass center of the posterior. Overall, CM had a higher maximum intensity in a mutual comparison to MAP for both real and synthetic data. This is particularly clear for the estimates with the elevated measurement noise (Figure 8). However, the location of the maximum was virtually the same. The marginal densities obtained show that, in the case of 1200 epochs, the maximum length of the 90% credibility interval for the marginal posterior’s mass center, conditional to the subjectively selected parameters, was in the range 0.9–1.4 and 1.2–2.3 mm, respectively, for the 1 and 2 mm FE mesh resolutions, thus matching the targeted range. The mutual differences in the median, for each coordinate direction, were less than 0.2 and 1.1 mm, respectively. As with the MAP, the spread of the CM that was obtained with the 2 mm FE mesh resolution varied more between the different reconstructions than in the case of the 1 mm mesh, while the maximal intensity of the CM was observed to vary less than that of the MAP. The marginal densities are clearly more spread out in the case of the elevated noise (Figure 8), i.e., with 300 and 100 epochs.

#### 3.1.4. Thalamic Component

The results for reconstructing the thalamic activity in the case of the sparse source space can be found in Figure 7, Figure 8 and Figure 9. Overall, when compared to the estimates obtained for the 3b area, the thalamic activity is less well-localized in its *a priori* expected location, which we expect to be primarily the ventral posterolateral nucleus [21] of the left thalamus contralateral to the stimulation side. This can be observed in the form of a greater variation between the estimates that were obtained for the thalamic component when compared to the 3b source localization. Some of these estimates were spread over the brainstem as well as the thalamic area [52,54]. Based on Figure 7, EEG has a greater tendency to find the deep activity than MEG. The CM estimate that is obtained with IG hyperprior is similar for both EEG and MEG, which suggests that the posterior exploration technique (here, MAP or CM) had a significant effect on detecting deep fluctuations. The reconstruction quality seems to be diminished when the noise level is elevated, as was the case for the 3b area (Figure 8). Nevertheless, the thalamic component was distinguishable with each noise level. Regarding the MNE and MCE estimates, MNE localized the deep activity, while MCE led to a strongly suppressed reconstruction (Figure 9).

### 3.2. Subject (II) and (III)

The results that were obtained for subjects (II) and (III) are visualized in Figure 10 in the case of the P20/N20 component and the EEG data. The results obtained show that the parameters used for subject (I) result in a largely appropriate reconstruction around the cortical (Brodmann 3b) and thalamic areas. The results are somewhat less focal than in the case of subject (I), which is obviously due to the measurement or head model creation process, while the activity is found with a uniform parameter choice for (I)–(III).

## 4. Discussion

In this proof-of-concept study, we proposed an approach for parametrizing the conditionally Gaussian hierarchical Bayesian model (CG-HBM) [7,8] and applied it to invert the P20/N20 response of the median nerve somatosensory evoked potentials and fields (SEP and SEF). We introduced an approach for parameter selection and analyzed its performance in source localization tests. The activity that corresponds to the P20/N20 response was detected in the Brodmann area 3b and the thalamus [21,22,52,53,55] while using both a dense and sparse source space [19], respectively. The source localization experiments were performed while using the Zeffiro interface (ZI) software tool [38].

In our approach, the shape and scale parameter determining the hyperprior and, thereby, also the conditionally Gaussian prior of the CG-HBM, were chosen based on optimization and *a priori* knowledge of the prior-over-measurement signal-to-noise ratio (PM-SNR), respectively. The shape parameter value β=3 was found to be close-to-optimal in minimizing the posterior differences between the gamma (G) and inverse gamma (IG) hyperprior (Appendix A). PM-SNR is a model specific constant which determines the scale parameter $\theta_{0}$ given the dimension and noise level of the forward model. The noise can consist of both a known and latent component. In this study, PM-SNR varied between 0 dB and 30 dB, depending on the assumption of the latent errors; the greater the errors, the higher the PM-SNR, i.e., the stronger the prior. As potential factors causing latent noise, we recognized (1) the forward modeling inaccuracies that are related to the measured data, e.g., the potential deviations of the conductivity distribution [46], which are absent for the simulated data, and also (2) the performance differences between the IAS and CM posterior estimation method. The value range applied in this study is in agreement with the Brainstorm software’s default MNE regularization value [27], which we estimated to match a PM-SNR of 10 dB with respect to EEG data.

The range that is proposed here is also supported by our recent studies [38] and [12]. In the first of these, IAS was shown to reconstruct a cortical epileptic (gyral) activity in EEG with both the G and IG hyperpriors, when the PM-SNR was set to 20 dB (following from $\theta_{0}=10^{-12}$, σ=0.03, N=100,000). In the other study, a scale parameter range from $10^{-10}$ to $10^{-8}$ was found to be applicable for IAS MAP estimation of numerically simulated deep activity with the IG hyperprior and a sparse source space (*N* from 100 to 400), converting to PM-SNR of 30 dB and 20 dB for $\theta_{0}=10^{-8},N\;=100$ and $\theta_{0}=10^{-10},N\;=400$, respectively.

The results obtained suggest that PM-SNR might also be applicable with elevated measurement noise levels, i.e., fewer averaged epochs, as both the 3b and thalamic activity components were found to be detectable in the cases of +6 dB and +10 dB noise. This result cannot be generalized, because the distinguishability of the responses is not obvious with fewer than the recommended minimum number of averaged epochs, which is 1000–4000 for SEPs [16,17,18] regarding the investigated 20 ms latency. Nevertheless, a lower number of averaged trials can be relevant in other EEG and MEG applications. For example, in [38], the reconstructions obtained with CG-HBM correspond to 58 averaged epileptiform discharges.

Comparing the performance of IAS obtained with the proposed settings and with those corresponding to MNE and MCE suggests that the present parametrization of CG-HBM can be related to, and also explain the performance of, classical regularization approaches. MNE was shown to find the thalamic activity with the selected PM-SNR, while it led to a visually less focal estimate in the 3b area than CG-HBM. In the case of β=1.5, MCE was observed to result in a suppressed deep activity, which suggested that the choice β=3 is advantageous with respect to MAP estimation with the G hyperprior. In particular, it seems that, with β=3, both the G and IG hyperpriors can find the deep source, supporting our approach of selecting β, so that it minimizes the difference between the hyperpriors. This difference is obvious, when β=1.5, which is, when the posterior maximizer of the prior variance θ will be zero for G and $\theta_{0}/3$ for IG (see Equation (A1)), suggesting that any weakly distinguishable (close-to-zero) fluctuations, especially the deep ones, will be suppressed by G.

The estimates that were obtained for the deep part varied more than those for the 3b area. This was expected, since the accuracy of both EEG and MEG is known to be limited with regard to far-field activity. Indeed, it has only recently been proposed that the localization of the deep sources is feasible based on non-invasive measurements [56,57]. In order to obtain an appropriate reconstruction for the thalamus, we applied a sparse source space, as it has recently been suggested that this can improve the detectability of the deep components [19]. Based on the results, it is obvious that the method applied in the posterior exploration has a major effect on the deep part of the reconstruction. As indicated by the numerical results of [7], the CM provided a more focal estimate for the thalamic activity than MAP in the case of MEG, which, in turn, is generally regarded as having less advantageous modality for depth-localization than EEG [10].

While the activity of the thalamus is generally known to overlap with that of the 3b area [21,22,53], there is less exact knowledge about the deep response network compared to that of the cortical one, and its activity varies subject-wise. For example, in [53], the activity that corrresponds to the P20/N20 peak was exclusively limited to the 3b area in ten subjects, while the thalamus was only found to be additionally activated in two subjects. In contrast, the somatosensory 3b component, i.e., the first cortical contribution in the median nerve SEP/SEF, is known to be well-localized in the posterior wall of the sulcus, while the gyral components will be visible a few milliseconds after 20 ms [21,52,58]. In order to obtain the best possible source localization outcome for both the superficial and deep areas, CG-HBM can be adapted to utilize multiple source space densities in finding a single reconstruction. An example of such a method is the recently proposed randomized multiresolution scanning (RAMUS) algorithm [12], which finds a reconstruction without imposing any restrictions regarding active brain areas or the source depth. The present restrictions (ROIs) have been introduced, as, here, our focus is on CG-HBM as a superclass of methods, rather than on the individual reconstruction techniques originating from it. That is why, here, we have restricted the number of moving parameters, other than the ones describing the statistical model.

Based on the present results, especially the position difference with respect to the mass center, it seems that a source localization accuracy of around 4 mm could be achieved in the 3b area. This coincides with the maximal spatial accuracy found for the MEG, i.e., 2–4 mm for the superficial areas [48,49], and even surpasses that of EEG, whose accuracy for superior locations was estimated to be, on average, approximately 9 mm [22,50,51]. A significant factor affecting the accuracy of EEG is the uncertainty related to the conductivity distribution [46,59]. However, while taking the total estimated 32–116 mm${}^{2}$ areal spread of the estimates obtained for the 3b area into account, the accuracy found here does not exceed the suggested maximal accuracy limits. The spread of the estimates arises from the current numerical framework as the maximal achievable focality without a potential numerical bias. The relationship between the estimates found and the actual physiological spread of the source is not evaluated here and it would necessitate further work.

CG-HBM might be advantageous as a statistical model for obtaining robust sampling-based inverse estimates, as suggested by the present results. When the parameters are chosen appropriately, the sampler-based approach seems to provide a robust technique for estimating the marginal posterior and the CM, giving information about the posterior distribution, shape and structure. Here, it allowed us to adapt it according to the underlying numerical model and geometry, whereas the IAS MAP estimation technique alone did not completely reveal the posterior shape, thereby increasing the risk of obtaining, e.g., overly focal estimates. It also seems that estimating the CM via a sampling approach and defining a ROI for the sampler is beneficial with respect to the distinguishability of the activity obtained. Because the IAS MAP estimation technique can be associated with many classical regularization methods, including MNE and MCE [7], sampling-based CG-HBM can also be seen as a potential way to enhance the outcome that was obtained with these classical methods.

The present forward simulation approach was found to perform adequately with both 1 and 2 mm resolution. Agreeing with the existing knowledge of physiologically accurate volumetric head modeling and forward simulation [32,60], the FE mesh resolution of 1 mm was observed to be advantageous for obtaining a satisfactorily consistent reconstruction quality. The present GPU-based approach to the forward simulation was found to be essential in order to achieve a suitably short computation time for the 1 mm mesh generation and lead field matrix evaluation processes with our ZI implementation. The approach of finding the reconstruction while using Cartesian source orientations was found to be suitable in the present modeling context, since it allows slight orientation changes during the source localization process, thereby resulting in a smooth posterior distribution. However, ZI also allows the normal orientations for cortical areas (Section 1) to be applied directly, as the differences between the directly normal and present normally-projected reconstructions seem minor.

More work will need to be done in order to optimize the outcome of CG-HBM for a given subject and dataset, as the present results mainly provide a proof-of-concept for a potentially applicable parametrization together with rough estimates for the parameter ranges. Therefore, an important objective of any future work will be to apply the present hyperprior parametrization technique for more datasets, e.g., including temporal correlations and combined E/MEG data, (see [12] for preliminary numerical simulation results, and subjects), in order to learn more about the practical localization capability of the CG-HBM. Potential directions for the development of mathematical method include, e.g., incorporating physiological knowledge to the hyperprior [61,62] and/or the source space that can be adapted to the properties of the active neural tissue: e.g., many brain structures have the primary current density of approximately 1 nAm/mm^2^ when active [63]. The development of source localization techniques for the multiple resolution levels [12], e.g., a full MCMC sampler implementation, provides a further potential application for the current parametrization approach. Finally, while our present focus is on CG-HBM and the corresponding reconstruction methods, i.e., IAS, MCMC sampling, MCE, and MNE, further research would be necessary in order to relate the performance of CG-HBM with that of the most promising alternative methods that do not belong to the CG-HBM superclass, such as the beamformer techniques [64,65,66].

## Figures and Tables

**Table 1:** The values of the prior-over-measurement signal-to-noise ratio (PM-SNR) for different reconstructions specified by the type of data (Measured/Synthetic), measurement modality (EEG/MEG), estimate (MAP/CM), and the hyperprior (G/IG). The source-wise $\theta_{0}$-value that was used for the dense and sparse source distribution corresponds to the total scale. PM-SNR can be associated with the *a priori* assumption on the latent noise strength, i.e., dB(s) (Section 2.1.4). When PM-SNR is 0 dB, the weight of the prior matches the *a priori* known noise level of 3%.

Data	Modality	Estimate	Hyp.	PM-SNR (dB)	Sparse $\theta_{0}$	Dense $\theta_{0}$
Meas.	EEG	MAP	G	20	$10^{-10}$	$10^{-12}$
			IG	30	$10^{-9}$	$10^{-11}$
		CM	G	20	$10^{-10}$	$10^{-12}$
			IG	20	$10^{-10}$	$10^{-12}$
	MEG	MAP	G	20	$10^{-10}$	$10^{-12}$
			IG	30	$10^{-9}$	$10^{-11}$
		CM	G	20	$10^{-10}$	$10^{-12}$
			IG	20	$10^{-10}$	$10^{-12}$
Synth.	EEG	MAP	G	20	$10^{-10}$	$10^{-12}$
			IG	30	$10^{-9}$	$10^{-11}$
		CM	G	0	$10^{-12}$	$10^{-14}$
			IG	0	$10^{-12}$	$10^{-14}$
	MEG	MAP	G	30	$10^{-9}$	$10^{-11}$
			IG	30	$10^{-9}$	$10^{-11}$
		CM	G	0	$10^{-12}$	$10^{-14}$
			IG	0	$10^{-12}$	$10^{-14}$

**Table 2:** Subject (I). The spread of the current distribution reconstructed in the Brodmann area 3b together with the position and orientation difference between the synthetic and reconstructed source. The position and orientation of the reconstruction was obtained from its center of mass in the spherical ROI. The MAP estimates were obtained without restricting the source space. In the case of CM, the reconstruction process was limited to ROI.

FE				MAP			Spread	Orientation	Position
Mesh	Data	Model	Data	/CM	Space	Hyper.	(mm${}^{2}$)	Δ (deg)	Δ (mm)
1 mm	Meas.	HBM	EEG	MAP	Global	G	44.7	9.7	3.4
					Global	IG	43.9	9.5	3.4
				CM	ROI	G	71.8	13.3	3.8
					ROI	IG	37.7	14.4	3.8
			MEG	MAP	Global	G	32.0	10.8	3.4
					Global	IG	32.2	10.8	3.4
				CM	ROI	G	48.8	11.7	3.7
					ROI	IG	42.0	12.5	3.8
	Synth.		EEG	MAP	Global	G	42.0	5.8	3.5
					Global	IG	40.3	5.7	3.5
				CM	ROI	G	64.8	11.5	3.7
					ROI	IG	69.9	12.1	3.7
			MEG	MAP	Global	G	22.0	12.8	3.5
					Global	IG	13.7	14.3	3.5
				CM	ROI	G	52.5	12.8	3.7
					ROI	IG	52.6	12.6	3.7
		+6 dB	EEG	MAP	Global	G	43.2	10.3	3.4
				CM	ROI	G	68.9	14.8	3.7
		+10 dB		MAP	Global	G	26.8	11.1	3.4
				CM	ROI	G	40.2	17.2	3.7
		Regul.	EEG	MNE	Global	G	38.1	9.2	3.4
				MCE	Global	G	12	8.8	3.6
2 mm	Meas.		EEG	MAP	Global	G	34.8	9.7	3.4
					Global	IG	30.9	9.7	3.4
				CM	ROI	G	43.4	10.8	3.5
					ROI	IG	67.7	7.0	3.4
			MEG	MAP	Global	G	87.9	11.5	3.5
					Global	IG	87.9	11.5	3.5
				CM	ROI	G	99.4	15.8	3.5
					ROI	IG	115.9	15.6	3.5
	Synth.		EEG	MAP	Global	G	64.2	6.7	3.3
					Global	IG	64.2	6.7	3.3
				CM	ROI	G	39.1	13.7	3.5
					ROI	IG	8.3	14.2	3.3
			MEG	MAP	Global	G	60.2	10.2	3.5
					Global	IG	51.0	10.0	3.5
				CM	ROI	G	109.0	16.1	3.5
					ROI	IG	110.2	16.1	3.5

**Figure A1:**
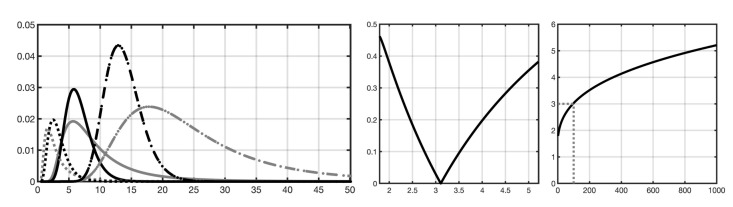
(**Left**) The *i*-th component of the conditional posterior $p(\theta_{i}|x_{i})$ optimized with respect to the shape parameter β so that ${\tilde{\theta }}_{i,\max}^{\left(G\right)}\approx c\;{\tilde{\theta }}_{i,\max}^{\left(\mathrm{IG}\right)}$ close to the point of linearization $x_{i}^{2}=\alpha \theta_{0}/\left(\beta -1\right)$ with some constant *c* (Appendix A). The gray curves correspond to IG and the black ones to G hyperprior. The solid, dashed and dash-dotted curves have been obtained with α=100, α=10 and α=1000, respectively. In each case the scale parameter is $\theta_{0}=1$. The maximizers match approximately, i.e., c≈1, when α=100 (solid) and the optimizer is β≈3. (**Center**) The relative scale deviation |c−1|/c w.r.t. c=1 as a function of the optimizer β, vanishing when β≈3. (**Right**) The optimizer β as the function of α.

**Figure A2:**
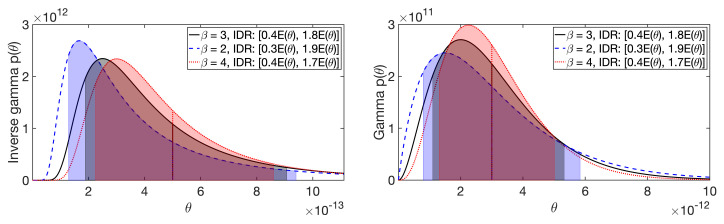
The effect of the shape parameter on the IG (**left**) and G (**right**) hyperprior. The black (solid), shows the hyperprior density p(θ) for β=3 and $\theta_{0}=$ 1 × 10^−12^ (see Table 1). The blue (dashed) and red (dotted) curves correspond to the same expectation E(θ) with β=2 and β=4, respectively. The greater the weight of the tail is, the smaller the shape parameter is. This is shown by the colored interdecile range (IDR), i.e., the interval between 10 and 90% quantile, which is presented here with respect to E(θ). The colors correspond to those of the curves.

## Appendix A. Shape Parameter Optimization

In HBM, the *i*-th entry x, is assumed to have an independent zero mean Gaussian distribution with variance $\theta_{i}$ determined by the G or the IG hyperprior. Given a shape and scale parameter β≥3/2 and $\theta_{0}>0$, the G and IG are maximized at the points $\theta_{i,\max}^{\left(G\right)\;}=\left(\beta -1\right)\theta_{0}$ and $\theta_{i,\max}^{\left(\mathrm{IG}\right)}=\theta_{0}/\left(\beta +1\right)$ and the mean is at $\theta_{i,\mathrm{mean}}^{\left(G\right)}=\beta \theta_{0}$ and $\theta_{i,\mathrm{mean}}^{\left(\mathrm{IG}\right)}=\theta_{0}/\left(\beta -1\right)$, respectively. Assuming that $\theta \ll \beta \ll 1/\theta_{0}$, the magnitude of these follows essentially from that of $\theta_{0}$. That is, the scale parameter sets the initial informativeness of the prior or the initial sensitivity of the hypermodel to localized brain activity. Generally, the smaller the value, the less informative is the prior, whose effect is slighter than the likelihood.

Given the *i*-th entry $x_{i}$, the maximizer of the joint prior p(θ,x) and of the conditional posterior p(θ∣x) w.r.t. θ, independent of the measurements y [7], is of the form

(A1) $${\tilde{\theta }}_{i,\max}^{\left(G\right)}=\frac{1}{2}\theta_{0}\left(\beta -\frac{3}{2}\right)+\frac{1}{2}\theta_{0}\sqrt{{\left(\beta -\frac{3}{2}\right)}^{2}+\frac{2x_{i}^{2}}{\theta_{0}}}\;\mathrm{and}\;{\tilde{\theta }}_{i,\max}^{\left(\mathrm{IG}\right)}=\frac{1}{\beta +\frac{3}{2}}\left(\theta_{0}+\frac{x_{i}^{2}}{2}\right).$$

In order to obtain comparable results with G and IG hyperprior and, thereby, to avoid any depth-localization bias of the G hyperprior [7,12], we optimize β so that the corresponding conditional posterior distributions are locally equally sensitive to the increment of $x_{i}^{2}$. That is, we optimize the shape parameter so that ${\tilde{\theta }}_{i,\max}^{\left(G\right)}\approx c\;{\tilde{\theta }}_{i,\max}^{\left(\mathrm{IG}\right)}$ locally with some constant *c* corresponding to a given $x_{i}^{2}$. We require that, following from the form ${\tilde{\theta }}_{i,\max}^{\left(\mathrm{IG}\right)}\left(s,\beta \right)=\nu +\nu s$, $\nu =\theta_{0}/\left(\beta +3/2\right)$, $s=x_{i}^{2}/\left(2\theta_{0}\right)$, there is a match between the zeroth and first-order coefficient of the first-order Taylor polynomial $F\left(s,\beta \right)\approx F\left(s_{0},\beta \right)+\frac{\partial F(s_{0},\beta )}{\partial s}\left(s-s_{0}\right)$ with $F\left(s,\beta \right)={\tilde{\theta }}_{i,\max}^{\left(G\right)}\left(s,\beta \right)$, resulting in the equation

(A2) $$F\left(s_{0},\beta \right)-\frac{\partial F(s_{0},\beta )}{\partial s}\left(s_{0}+1\right)=0$$

with respect to β. Furthermore, we set the point of linearization proportional to the mean of the IG hyperprior, i.e., $s_{0}=\alpha /\left[2\left(\beta -1\right)\right]$ or $x_{i}^{2}=\alpha \;\theta_{i,\mathrm{mean}}^{\left(\mathrm{IG}\right)}$. That is, $x_{i}^{2}$ is assumed to be proportional to the expected variance of the conditional Gaussian prior. We make this choice, since a zero-mean Gaussian density is invariant with respect to a scale proportional to its variance.

(Figure A1) illustrates the results obtained by solving the Equation (A2) numerically with respect to β for each integer α from α=1 to α=1000. For α=100, the solution and the corresponding scaling constant satisfy β≈3 and c≈1, meaning that $\theta_{i,\mathrm{mean}}^{\left(\mathrm{IG}\right)}\approx \theta_{i,\mathrm{mean}}^{\left(G\right)}$ at the point of linearization. For this match, we interpret β=3 as a close-to-optimal shape parameter value to minimize the differences between the G and IG hyperprior. As can be observed from (Figure A1), an IG hyperprior generally leads to a heavier-tailed conditional posterior distribution than G at the point of linearization, meaning that, in principle, it allows a higher probability for outliers such as focal activity spots.

## Appendix B. Effect of the Shape Parameter

The effect of the shape parameter on the hyperprior is visualized in Figure A2. The weight of the tail is the greater the smaller the shape parameter which is shown by the interdecile range (the interval between 10 and 90% quantile).

## References

[1] Hämäläinen M., Hari R., Ilmoniemi R.J., Knuutila J., Lounasmaa O.V. (1993). Magnetoencephalography— Theory, instrumentation, and applications to invasive studies of the working human brain. Rev. Mod. Phys..

[2] Niedermeyer E., da Silva F.L. (2004). Electroencephalography: Basic Principles, Clinical Applications, and Related Fields.

[3] Brette R., Destexhe A. (2012). Handbook of Neural Activity Measurement.

[4] Wipf D., Nagarajan S. (2009). A unified Bayesian framework for MEG/EEG source imaging. NeuroImage.

[5] Friston K., Harrison L., Daunizeau J., kiebel S., Phillips C., Trujillo-Barreto N., Henson R., Flandin G., Mattout J. (2008). Multiple sparse priors for the M/EEG inverse problem. NeuroImage.

[6] Sato M., Yoshioka T., Kajihara S., Toyama K., Goda N., Doya K., Kawato M. (2004). Hierarchical bayesian estimation for MEG inverse problem. NeuroImage.

[7] Calvetti D., Hakula H., Pursiainen S., Somersalo E. (2009). Conditionally Gaussian hypermodels for cerebral source localization. SIAM J. Imaging Sci..

[8] Lucka F., Pursiainen S., Burger M., Wolters C.H. (2012). Hierarchical bayesian inference for the EEG inverse problem using realistic FE head models: Depth localization and source separation for focal primary currents. NeuroImage.

[9] Ahlfors S.P., Han J., Belliveau J.W., Hämäläinen M.S. (2010). Sensitivity of MEG and EEG to source orientation. Brain Topogr..

[10] Hari R., Baillet S., Barnes G., Burgess R., Forss N., Gross J., Hämäläinen M., Jensen O., Kakigi R., Mauguière F. (2018). IFCN-endorsed practical guidelines for clinical magnetoencephalography (MEG). Clin. Neurophysiol..

[11] O’Hagan A., Forster J.J. (2004). Kendall’s Advanced Theory of Statistics.

[12] Rezaei A., Koulouri A., Pursiainen S. (2020). Randomized multiresolution scanning in focal and fast E/MEG sensing of brain activity with a variable depth. Brain Topogr..

[13] Rampp S., Stefan H., Wu X., Kaltenhäuser M., Maess B., Schmitt F.C., Wolters C.H., Hamer H., Kasper B.S., Schwab S. (2019). Magnetoencephalography for epileptic focus localization in a series of 1000 cases. Brain.

[14] Foley E., Cerquiglini A., Cavanna A., Nakubulwa M.A., Furlong P.L., Witton C., Seri S. (2014). Magnetoencephalography in the study of epilepsy and consciousness. Epilepsy Behav..

[15] Foley E., Cross J.H., Thai N.J., Walsh A.R., Bill P., Furlong P., Wood A.G., Cerquiglini A., Seri S. (2019). MEG assessment of expressive language in children evaluated for epilepsy surgery. Brain Topogr..

[16] (2006). American Clinical Neurophysiology Society and others, Guideline 9A: Guidelines on evoked potentials. J. Clin. Neurophysiol. Off. Publ. Am. Electroencephalogr. Soc..

[17] Cruccu G., Aminoff M.J., Curio G., Guerit J.M., Kakigi R.R., Mauguiere F., Rossini P.M., Treede R.D., Garcia-Larrea L. (2008). Recommendations for the clinical use of somatosensory-evoked potentials. Clin. Neurophysiol..

[18] Desmedt J.E., Guy C. (1980). Somatosensory evoked potentials to finger stimulation in healthy octogenarians and in young adults: Wave forms, scalp topography and transit times of pariental and frontal components. Electroencephalogr. Clin. Neurophysiol..

[19] Krishnaswamy P., Obregon-Henao G., Ahveninen J., Khan S., Babadi B., Iglesias J.E., Hämäläinen M.S., Purdon P.L. (2017). Sparsity enables estimation of both subcortical and cortical activity from MEG and EEG. Proc. Natl. Acad. Sci. USA.

[20] Samuelsson J.G., khan S., Sundaram P., Peled N., Hämäläinen M.S. (2019). Cortical Signal Suppression (CSS) for detection of subcortical activity using MEG and EEG. Brain Topogr..

[21] Haueisen J., Leistritz L., Süsse T., Curio G., Witte H. (2007). Identifying mutual information transfer in the brain with differential-algebraic modeling: Evidence for fast oscillatory coupling between cortical somatosensory areas 3b and 1. NeuroImage.

[22] Buchner H., Fuchs M., Wischmann H.A., Dössel O., Ludwig I., Knepper A., Berg P. (1994). Source analysis of median nerve and finger stimulated somatosensory evoked potentials: Multichannel simultaneous recording of electric and magnetic fields combined with 3D-MR tomography. Brain Topogr..

[23] Hari R., Puce A. (2017). MEG-EEG Primer.

[24] Allison T., Wood C.C., McCarthy G., Spencer D.D. (1991). Cortical somatosensory evoked potentials. ii. effects of excision of somatosensory or motor cortex in humans and monkeys. J. Neurophysiol..

[25] Fuchs M., Wagner M., Wischmann H.A., Köhler T., Theißen A., Drenckhahn R., Buchner H. (1998). Improving source reconstructions by combining bioelectric and biomagnetic data. Clin. Neurophysiol..

[26] Baillet S., Mosher J.C., Leahy R.M. (2001). Electromagnetic brain mapping. IEEE Signal Process. Mag..

[27] (2020). Tutorial 22: Source Estimation. https://neuroimage.usc.edu/brainstorm/Tutorials/SourceEstimation.

[28] Kaipio J.P., Somersalo E. (2004). Statistical and Computational Methods for Inverse Problems.

[29] Nummenmaa A., Auranen T., Hämäläinen M.S., Jääskeläinen I.P., Lampinen J., Sams M., Vehtari A. (2007). Hierarchical Bayesian estimates of distributed MEG sources: Theoretical aspects and comparison of variational and MCMC methods. NeuroImage.

[30] Sommariva S., Sorrentino A. (2014). Sequential Monte Carlo samplers for semi-linear inverse problems and application to magnetoencephalography. Inverse Probl..

[31] Pursiainen S., Vorwerk J., Wolters C.H. (2016). Electroencephalography (EEG) forward modeling via H(div) finite element sources with focal interpolation. Phys. Med. Biol..

[32] De Munck J.C., Wolters C.H., Clerc M., Brette R., Destexhe A. (2012). EEG & MEG forward modeling. Handbook of Neural Activity Measurement.

[33] Braess D. (2001). Finite Elements.

[34] Haueisen J., Tuch D.S., Ramon C., Schimpf P.H., Wedeen V.J., George J.S., Belliveau J.W. (2002). The influence of brain tissue anisotropy on human EEG and MEG. NeuroImage.

[35] Ramon C., Schimpf P., Haueisen J. (2006). Influence of head models on EEG simulations and inverse source localizations. Biomed. Eng. Online.

[36] Beltrachini L. (2018). Sensitivity of the projected subtraction approach to mesh degeneracies and its impact on the forward problem in EEG. IEEE Trans. Biomed. Eng..

[37] Vorwerk J., Cho J.H., Rampp S., Hamer H., Knösche T.R., Wolters C.H. (2014). A guideline for head volume conductor modeling in EEG and MEG. NeuroImage.

[38] He Q., Rezaei A., Pursiainen S. (2019). Zeffiro user interface for electromagnetic brain imaging: A GPU accelerated fem tool for forward and inverse computations in Matlab. Neuroinformatics.

[39] Uutela K., Hämäläinen M., Somersalo E. (1999). Visualization of magnetoencephalographic data using minimum current estimates. NeuroImage.

[40] Miinalainen T., Rezaei A., Us D., Nüßing A., Engwer C., Wolters C.H., Pursiainen S. (2019). A realistic, accurate and fast source modeling approach for the EEG forward problem. NeuroImage.

[41] Pursiainen S. (2012). Raviart–Thomas-type sources adapted to applied EEG and MEG: Implementation and results. Inverse Probl..

[42] Schmidt D.M., George J.S., Wood C.C. (1999). Bayesian inference applied to the electromagnetic inverse problem. Hum. Brain Mapp..

[43] Calvetti D., Somersalo E. (2007). A Gaussian hypermodel to recover blocky objects. Inverse Probl..

[44] Ahlfors S.P., Hämäläinen M., Sharon D., Ishitobi M., Vaina L.M., Stufflebeam S.M. (2009). Mapping the signal-to-noise-ratios of cortical sources in magnetoencephalography and electroencephalography. Hum. Brain Mapp..

[45] Wolters C.H., Lew S., Macleod R.S., Hämäläinen M. (2010). Combined EEG/MEG source analysis using calibrated finite element head models. Biomed. Tech. Eng. Rostock. Ger. Walter Gruyter.

[46] Antonakakis M., Schrader S., Wollbrink A., Oostenveld R., Rampp S., Haueisen J., Wolters C.H. (2019). The effect of stimulation type, head modeling, and combined EEG and meg on the source reconstruction of the somatosensory P20/N20 component. Hum. Brain Mapp..

[47] Pursiainen S., Lucka F., Wolters C.H. (2012). Complete electrode model in EEG: Relationship and differences to the point electrode model. Phys. Med. Biol..

[48] Tarkiainen A., Liljeström M., Seppä M., Salmelin R. (2003). The 3D topography of MEG source localization accuracy: Effects of conductor model and noise. Clin. Neurophysiol..

[49] Cohen D., Cuffin B.N. (1991). EEG versus MEG localization accuracy: Theory and experiment. Brain Topogr..

[50] Cuffin B.N., Schomer D.L., Ives J.R., Blume H. (2001). Experimental tests of EEG source localization accuracy in realistically shaped head models. Clin. Neurophysiol..

[51] Cuffin B.N., Schomer D.L., Ives J.R., Blume H. (2001). Experimental tests of EEG source localization accuracy in spherical head models. Clin. Neurophysiol..

[52] Buchner H., Adams L., Müller A., Ludwig I., Knepper A., Thron A., Niemann K., Scherg M. (1995). Somatotopy of human hand somatosensory cortex revealed by dipole source analysis of early somatosensory evoked potentials and 3D-NMR tomography. Electroencephalogr. Clin. Neurophysiol. Potentials Sect..

[53] Götz T., Huonker R., Witte O.W., Haueisen J. (2014). Thalamocortical impulse propagation and information transfer in EEG and MEG. J. Clin. Neurophysiol..

[54] Buchner H., Adams L., Knepper A., Rüger R., Laborde G., Gilsbach J.M., Ludwig I., Reul J., Scherg M. (1994). Preoperative localization of the central sulcus by dipole source analysis of early somatosensory evoked potentials and three-dimensional magnetic resonance imaging. J. Neurosurg..

[55] Buchner H., Knoll G., Fuchs M., Rienäcker A., Beckmann R., Wagner M., Silny J., Pesch J. (1997). Invers localization of electric dipole current sources in finite element models of the human head. Electroencephalogr. Clin. Neurophysiol..

[56] Seeber M., Cantonas L.M., Hoevels M., Sesia T., Visser-Vandewalle V., Michel C.M. (2019). Subcortical electrophysiological activity is detectable with high-density EEG source imaging. Nat. Commun..

[57] Pizzo F., Roehri N., Villalon S.M., Trébuchon A., Chen S., Lagarde S., Carron R., Gavaret M., Giusiano B., McGonigal A. (2019). Deep brain activities can be detected with magnetoencephalography. Nat. Commun..

[58] Papadelis C., Eickhoff S.B., Zilles K., Ioannides A.A. (2011). BA3b and BA1 activate in a serial fashion after median nerve stimulation: Direct evidence from combining source analysis of evoked fields and cytoarchitectonic probabilistic maps. Neuroimage.

[59] Wang G., Yang L., Worrell G., He B. The relationship between conductivity uncertainties and EEG source localization accuracy. Proceedings of the 2009 Annual International Conference of the IEEE Engineering in Medicine and Biology Society.

[60] Vorwerk J., Aydin Ü., Wolters C.H., Butson C.R. (2019). Influence of head tissue conductivity uncertainties on EEG dipole reconstruction. Front. Neurosci..

[61] Calvetti D., Pascarella A., Pitolli F., Somersalo E., Vantaggi B. (2015). A hierarchical Krylov–Bayes iterative inverse solver for MEG with physiological preconditioning. Inverse Probl..

[62] Calvetti D., Pascarella A., Pitolli F., Somersalo E., Vantaggi B. (2018). Brain activity mapping from MEG data via a hierarchical Bayesian algorithm with automatic depth weighting. Brain Topogr..

[63] Murakami S., Okada Y. (2015). Invariance in current dipole moment density across brain structures and species: Physiological constraint for neuroimaging. Neuroimage.

[64] Steinsträter O., Sillekens S., Junghoefer M., Burger M., Wolters C.H. (2010). Sensitivity of beamformer source analysis to deficiencies in forward modeling. Hum. Brain Mapp..

[65] Sekihara K., Sahani M., Nagarajan S.S. (2005). Localization bias and spatial resolution of adaptive and non-adaptive spatial filters for MEG source reconstruction. Neuroimage.

[66] Neugebauer F., Möddel G., Rampp S., Burger M., Wolters C.H. (2007). The effect of head model simplification on beamformer source localization. Front. Neurosci..

